# Mothers and Fathers with Binge Eating Disorder and Their 18–36 Months Old Children: A Longitudinal Study on Parent–Infant Interactions and Offspring’s Emotional–Behavioral Profiles

**DOI:** 10.3389/fpsyg.2016.00580

**Published:** 2016-04-25

**Authors:** Silvia Cimino, Luca Cerniglia, Alessio Porreca, Alessandra Simonelli, Lucia Ronconi, Giulia Ballarotto

**Affiliations:** ^1^Department of Dynamic and Clinical Psychology, Sapienza – University of RomeRome, Italy; ^2^Department of Psychology, International Telematic University UninettunoRome, Italy; ^3^Department of Developmental and Social Psychology, University of PaduaPadua, Italy; ^4^Department of General Psychology, University of PaduaPadua, Italy

**Keywords:** Binge Eating Disorder, parent–infant interactions, emotional–behavioral profiles, child-development, fathers

## Abstract

Maternal Binge Eating Disorder (BED) has been suggested to be associated with poor parent–infant interactions during feeding and with children’s emotional and behavioral problems during infancy (Blissett and Haycraft, 2011). The role of fathers has received increasing consideration in recent years, yet the research has not focused on interactional patterns between fathers with BED and their children. The present study aimed to longitudinally investigate the influence of BED diagnosis, in one or both parents, on parent–infant feeding interactions and on children’s emotional–behavioral functioning. 612 subjects (408 parents; 204 children), recruited in mental health services and pre-schools in Central Italy, were divided into four groups: Group 1 included families with both parents diagnosed with BED, Group 2 and 3 included families with one parent diagnosed with BED, Group 0 was a healthy control. The assessment took place at T1 (18 months of age of children) and T2 (36 months of age of children): feeding interactions were assessed through the Scale for the Assessment of Feeding Interactions (SVIA) while child emotional–behavioral functioning was evaluated with the Child Behavior Check-List (CBCL). When compared to healthy controls, the groups with one or both parents diagnosed with BED showed higher scores on the SVIA and on the CBCL internalizing and externalizing scales, indicating poorer adult–child feeding interactions and higher emotional–behavioral difficulties. A direct influence of parental psychiatric diagnosis on the quality of mother–infant and father–infant interactions was also found, both at T1 and T2. Moreover, dyadic feeding interactions mediated the influence of parental diagnosis on children’s psychological functioning. The presence of BED diagnosis in one or both parents seems to influence the severity of maladaptive parent–infant exchanges during feeding and offspring’s emotional–behavioral problems over time, consequently affecting different areas of children’s psychological functioning. This is the first study to demonstrate the specific effects of maternal and paternal BED on infant development. These results could inform prevention and intervention programs in families with one or both parents diagnosed with BED.

## Introduction

Binge Eating Disorder (BED) has recently been included in the DSM-5 classification system (American Psychiatric Association, 2013) and its lifetime prevalence has been estimated ∼2% in adults. Although several authors have addressed BED clinical manifestations, its correlated psychopathological symptoms, and possible treatment approaches, there is a dearth of longitudinal research on mothers and fathers with BED and on the possible weight of this disorder on their children’s emotional–behavioral functioning in their first years of life (Cimino et al., 2015; de Barse et al., 2015). The Developmental Psychopathology theoretical framework considers psychopathology transmission from parents to children as mediated by individual and relational, genetic and environmental factors, and also emphasizes the role of the quality of parent–infant interactions in shaping offspring’s mental health (Bifulco et al., 2002; Davies and Cicchetti, 2004). Moreover, seminal studies of (Bifulco et al., 2014a,b) have shown that not only parental psychiatric disorders, but also other adverse attachment experiences may lead children to atypical development (Schimmenti and Bifulco, 2015). Strober et al. (2000) have demonstrated that children of parents with Eating Disorders are liable to homotypical or heterotypical syndromes, whereas other authors have underlined the association between maternal Anorexia and Bulimia (Taborelli et al., 2015) and offspring’s maladaptive psychological profiles. Though the role of maternal psychiatric disorders on offspring’s psychological functioning has been widely assessed (Teti et al., 1995; Riahi et al., 2012; Paciello et al., 2013; Tambelli et al., 2015a,b) and, more recently, paternal psychopathological risk has been also considered as an adjunct problematic factor associated with children’s internalizing and externalizing symptoms (Lamb, 2010; Cimino et al., 2013), only a few studies have focused on the observation of interactive patterns during feeding in families of children in their first 3 years of life where both parents were diagnosed for BED. The observation of children’s interactions with ED diagnosed parents has shown exchanges characterized by asynchrony, scarce involvement, and a lack of sharing positive affective bonds (Beebe et al., 2012). It has been suggested that the quality of these interactions may vary over time (end especially during the first 3 years of life of the child), and can improve due to parents’ adjustment to their offspring characteristics (e.g., child’s difficult temperament), increased family and/or marital support, remission of psychopathological symptoms in the parents, or it can worsen (e.g., for adjunct risk factors, inefficacy of psychological or psychiatric interventions, etc.). Neurobiological studies have also suggested that early disruptions of the mother–infant relationship may have a negative impact on offspring’s brain plasticity, with important implications for their psychopathology (Cirulli et al., 2003). Thus, it has been underlined that longitudinal studies are needed in this field and in samples with psychiatrically diagnosed parents to assess the stability and change both of the quality of parent–infant interactions and of their offspring’s psychological internalizing and externalizing difficulties (Halligan et al., 2013).

Notwithstanding the above studies, to our knowledge the specific weight of the quality of interactive exchanges during feeding between parents with BED and their offspring in predicting children’s psychopathology has not been specifically studied.

Based on the above premises, we recruited for a longitudinal study (Time 1: 18 months of age of the child; Time 2: 36 months of age of the child) a sample of families where both parents (Group 1), only the mother (Group 2), only the father (Group 3) were diagnosed for BED and a healthy control group (Group 0) aiming to:

(1) Assess mother–child’s and father–child’s interactions during feeding at T1 and T2, verifying possible significant differences between the four groups;
(2) Assess stability and change of internalizing and externalizing symptoms of the children at T1 and T2;
(3) Assess the possible role of the quality of parent–infant interactions during feeding in predicting offspring’s internalizing and externalizing symptoms in Groups 1–3.

## Materials and Methods

### Subjects and Procedure

The study involved 208 families (*N*_tot_ = 416) who attended, over a 1-year period, a network of public consultants in Central Italy for the assessment of BED in adults. We excluded families if the mother and the father were not personally handling personally the child’s care and nutrition (for example delegating the child’s feeding to grandparents because mothers and fathers are at work during the day; *N* = 32). In the remaining sample group (*N* = 176 mothers and *N* = 176 fathers), 162 mothers and 153 fathers were diagnosed with BED without comorbidity by psychiatrists from the various consultant offices, according to DSM-5 criteria (American Psychiatric Association, 2013). *N* = 21 parents received a different diagnosis (*N* = 8 anxiety disorder; *N* = 6 borderline personality disorder; *N* = 7 BED with a comorbid anxiety disorder) and were suggested to follow protocols that were not included in this study. *N* = 16 subjects were excluded from the study due to the following criteria: parents referred medical or psychiatric diagnosis of the child, parents and/or children were pursuing medication-based treatment, parents and/or children were pursuing psychiatric or psychological treatment. Three groups were composed on the basis of the presence of BED diagnosis in both parents (Group 1; parents: *N* = 102; offspring: *N* = 51), only in mothers (Group 2; parents: *N* = 104; offspring: *N* = 52), or only in fathers (Group 3; parents: *N* = 100; offspring: *N* = 50). This sample was paired with a healthy control (Group 0; parents: *N* = 102; offspring: *N* = 51), comparable for socio-demographic characteristics and randomly chosen among a wider sample recruited from collaborating primary schools in Central Italy. **Table 1** reports the characteristics of the participants. Each group was balanced with respect to the children’s gender and age. Most of the children were first-born (85%), and all were natural children of their parents. Ninety-one percent of children belonged to intact families.

**Table 1:** Characteristics of the subjects of the study at Time 1.

	Group 0	Group 1	Group 2	Goup 3
	
*N*	51	51	52	50
Children’s gender	24 (47,1%) m	27 (52,9%) m	25 (48,1%) m	26 (52%) m
	27 (52,9%) f	24 (47,1 %) f	27 (51,9%) f	24 (48%) f
Children’s age (months)	20.02 (2.86)	19.59 (2.40)	19.23 (2.30)	19.26 (2.28)
Mothers’ age (years)	32.47 (2.75)	33.04 (3.77)	32.33 (2.98)	32.16 (3.05)
Fathers’ age (years)	35.45 (4.75)	35.31 (5.07)	36.00 (4.36)	35.60 (4.81)

*Group 0, control; Group 1, both parents diagnosed with BED; Group 2, mother diagnosed with BED; Group 3, father diagnosed with BED.*

The groups were evaluated with the tools described below at two time points with an inter-evaluation interval of ∼18 months. The first time point (T1) was when the children were 18 months old, and the second time point (T2) was when they were 36 months old. The clinical equipe was composed of six psychologists within the public health care system specifically trained in the use of the tools used in the study. The research described here was approved by the Ethical Committee of the Psychology Faculty at Sapienza, University of Rome, before the start of the study and in accordance with the Declaration of Helsinki. Written informed consent was obtained from each of the study participants.

### Tools

Mother–infant and father–infant interactions during feeding were assessed through the Scale for the Assessment of Feeding Interactions (Scala di Valutazione Interazioni Alimentari – SVIA). The tool was administered separately for mother–child and father–child dyads during a main meal at their home. Moreover, parents completed the Child Behavior Checklist (CBCL 1½–5), described below, at T1, T2, and independently.

### Scale for the Assessment of Feeding Interactions (SVIA)

The SVIA is the Italian adaptation of the Feeding Scale (Chatoor et al., 1997) that can be applied to children between the ages of 12–36 months old. It measures interactive behaviors and identifies normal and/or risky relational modes between a parent and child during feeding exchanges (Lucarelli et al., 2002). Parent–infant interactions during feeding are recorded for at least 20 min, and then a wide range of interactive mother–infant behaviors are coded and evaluated. The SVIA consists of 41 items distributed among four subscales: (1) Parent’s affective states (index of the parent’s affective states); (2) Interactive conflict (index of interactions characterized by conflictual, non-collaborative, and non-empathetic communication); (3) Food refusal behavior (habits associated with challenged status regulation during meals and with limited food consumption); and (4) Dyad’s affective state (index of the extent to which the infant’s feeding patterns are, or are not, the result of an interactive regulation to which both partners contribute). The scores, measured on 4-point a Likert Scale ranging from 0 to 3 (none, a little, quite a bit, a lot). Inter-evaluator agreement for SVIA items is generally good to excellent (Pearson r values, 0.7–1.0 for group of 182 normal infants and 0.9–1.0 for a group of 182 infants with nutritional disorders). And the instrument shows good reliability, in terms of internal consistency (Cronbach’s α, 0.79–0.96).

### Child Behavior Check-List

The CBCL is a questionnaire filled out by parents and caregivers with the purpose of assessing the child’s abilities and his/her specific behavioral/emotional characteristics. The CBCL 1½–5 (Achenbach and Rescorla, 2001) is composed of 100 items that lead to two summary scales. The Internalizing Problems Scale consists of four syndrome subscales: Emotionally Reactive, Anxious/Depressed, Somatic Complaints, and Withdrawn. The Externalizing Problems Scale is composed of two syndrome subscales: Attention Problems and Aggressive Behavior. The CBCL 1½–5 has high test–retest reliability and high internal consistency (Achenbach and Rescorla, 2001). The criterion-related validity of both versions of the CBCL is supported by the ability of the CBCL’s quantitative scale scores to discriminate between demographically matched, referred, and non-referred children (Kim et al., 2012). In the present study, we used the Italian validated versions and the Italian cut-off values (Frigerio et al., 2006).

## Results

Data were analyzed using IBM SPSS statistics version 23 and LISREL 8.80 (Jöreskog and Sörbom, 2006). Both qualitative and quantitative analyses were performed on data obtained. The qualitative analyses were run using descriptive statistics (reliability of the measures, frequencies, mean scores and percentages). Mixed ANOVAs were conducted on data concerning the SVIA and the CBCL, considering the Group as the between-subjects factor (0 vs. 1 vs. 2 vs. 3) and Time as the within-subject factor (T1 vs. T2). Later, data were analyzed considering the presence/absence of maternal/paternal diagnosis of BED. In this case, Pearson’s product-moment correlation analysis was used to test the relationship between parental BED diagnoses, quality of feeding interactions and the presence of internalizing or externalizing symptoms during T2. Finally, Structural Equation Modeling (SEM) was used to test the causal assumptions made about the structural relations of the measures.

### Preliminary Analysis

In the preliminary analysis Cronbach’s alpha coefficient was used to assess the reliability of the instruments. A qualitative analysis was also run, using descriptive statistics (average scores, frequencies, and percentages).

#### Mother–Child and Father–Child Feeding Interactions

The Cronbach’s alpha coefficient indicated excellent reliability for the SVIA subscales concerning both mother–child (0.941 ≤ α ≤ 0.959) and father–child (0.945 ≤ α ≤ 0.963) feeding interactions. **Tables 2** and **3** report average scores and standard deviations of the SVIA subscales concerning, respectively, mother–child and father–child exchanges.

**Table 2:** Average scores and standard deviations of the SVIA subscales applied during mother–child feeding interactions.

		Group 0	Group 1	Group 2	Group 3
		
		*M* (*SD*)	*M* (*SD*)	*M* (*SD*)	*M* (*SD*)
Mother’s Affective state	T1	9.83 (4.52)	24.16 (2.01)	17.54 (5.83)	11.58 (4.05)
	T2	2.74 (1.14)	23.86 (2.55)	16.40 (3.65)	9.63 (3.02)
Interactive conflict	T1	7.97 (4.30)	22.14 (2.01)	16.60 (5.50)	10.76 (3.67)
	T2	2.44 (1.07)	21.24 (2.35)	15.24 (2.89)	10.43 (2.60)
Food refusal behavior	T1	5.24 (2.18)	13.02 (1.45)	9.68 (2.70)	5.94 (2.35)
	T2	1.47 (0.67)	12.14 (1.74)	9.03 (2.04)	5.39 (1.37)
Dyad’s Affective state	T1	4.30 (2.64)	15.13 (1.62)	9.95 (3.52)	6.35 (2.40)
	T2	1.47 (0.75)	13.70 (1.83)	9.12 (2.09)	5.70 (1.39)

*Group 0, control; Group 1, both parents diagnosed with BED; Group 2, mother diagnosed with BED; Group 3, father diagnosed with BED.*

**Table 3:** Average scores and standard deviations of the SVIA subscales applied during father–child feeding interactions.

		Group 0	Group 1	Group 2	Group 3
		
		*M* (*SD*)	*M* (*SD*)	*M* (*SD*)	*M* (*SD*)
Father’s Affective state	T1	9.84 (4.43)	24.85 (2.93)	12.15 (4.45)	18.58 (5.85)
	T2	2.78 (1.09)	22.87 (2.55)	9.92 (2.64)	20.16 (2.06)
Interactive conflict	T1	7.96 (4.28)	22.19 (2.47)	11.34 (4.26)	17.04 (5.09)
	T2	2.45 (1.06)	20.21 (2.40)	11.13 (2.36)	18.08 (1.94)
Food refusal behavior	T1	5.26 (2.20)	12.86 (1.85)	6.29 (2.51)	10.09 (2.79)
	T2	1.51 (0.62)	12.35 (1.90)	5.53 (1.43)	10.92 (1.23)
Dyad’s Affective state	T1	4.27 (2.60)	14.97 (1.75)	6.97 (2.40)	10.39 (3.48)
	T2	1.45 (0.70)	13.02 (1.80)	6.07 (1.37)	11.82 (1.05)

*Group 0, control; Group 1, both parents diagnosed with BED; Group 2, mother diagnosed with BED; Group 3, father diagnosed with BED.*

#### Child’s Internalizing and Externalizing Symptoms

The application of Cronbach’s alpha coefficient to the CBCL items indicated excellent reliability of the instrument both during T1 (α = 0.938) and T2 (α = 0.942). **Table 4** reports average scores and standard deviations for the CBCL summary scales concerning Externalizing and Internalizing problems. **Table 5** reports the distribution of the four groups of subjects in the ranges (Normative, Border, Clinical) yielded by the scoring procedure of the CBCL with respect to the summary scales.

**Table 4:** Average scores and standard deviations of the CBCL syndrome scales, the summary scales and of the CBCL total score.

		Group 0	Group 1	Group 2	Group 3
		
		*M* (*SD*)	*M* (*SD*)	*M* (*SD*)	*M* (*SD*)
Internalizing	T1	12.75 (9.82)	33.47 (5.18)	23.81 (6.97)	29.72 (10.54)
	T2	9.43 (4.00)	27.31 (6.83)	26.10 (6.85)	34.28 (5.29)
Externalizing	T1	8.45 (5.46)	22.20 (4.03)	12.19 (5.09)	19.40 (5.32)
	T2	5.47 (1.93)	22.61 (3.62)	15.98 (4.52)	21.74 (3.30)

*Group 0, control; Group 1, both parents diagnosed with BED; Group 2, mother diagnosed with BED; Group 3, father diagnosed with BED.*

**Table 5:** Distribution of the subjects within the Normative, the Border, and the Clinical range with respect to the CBCL summary scales.

		Internalizing		Externalizing	
		T1	T2	T1	T2
		
		*N* (%)	*N* (%)	*N* (%)	*N* (%)
**Group 0**	Norm	40 (78,4%)	51 (100%)	51 (100%)	51 (100%)
*N* = 51	Border	1 (2%)	–	–	–
	Clinical	10 (19,6%)	–	–	–
**Group 1**	Norm	–	8 (15,7%)	38 (74,5%)	38 (74,5%)
*N* = 51	Border	5 (9,8%)	8 (15,7%)	13 (25,5%)	13 (25,5%)
	Clinical	46 (90,2%)	35 (68,6%)	–	–
**Group 2**	Norm	14 (26,9%)	6 (11,5%)	51 (98%)	50 (96,2%)
*N* = 52	Border	20 (38,5%)	23 (44,2%)	1 (2%)	2 (3,8%)
	Clinical	18 (34,6%)	23 (44,2%)	–	–
**Group 3**	Norm	10 (20%)	–	45 (90%)	43 (86%)
*N* = 50	Border	2 (4%)	3 (6%)	5 (10%)	7 (14%)
	Clinical	38 (76%)	47 (94%)	–	–

*Group 0, control; Group 1, both parents diagnosed with BED; Group 2, mother diagnosed with BED; Group 3, father diagnosed with BED*.

As it is possible to see from the table, with respect to Internalizing symptoms, during T1 Group 1 showed the highest scores. During T2, instead, the highest scores on internalizing symptoms were the ones concerning Group 3 (the one with the father diagnosed with BED). When considering the change from T1 to T2, there was a slight decrease in Group 0, Group 1 and Group 2’s Internalizing symptoms, whereas Group 3 showed an increase in those scores. Moreover, with respect to Externalizing symptoms, Group 1 (where both parents were diagnosed with BED) showed higher scores both during T1 and T2. When considering the change between the two periods, it was possible to see a decrease in Group 0’s scores, while the groups characterized by one (Group 2 and 3) or both (Group 1) parents diagnosed with BED seemed to experience an increase in the perception of children’s externalizing symptoms.

### Mother–Child Feeding Interactions: Differences between Groups and Changes in Time

In order to investigate the presence of differences between the four groups in the quality of mother–child feeding interactions and the presence of changes in time of such interactions, a mixed ANOVA was conducted on the data collected, with Group as between-subjects factor (0 vs. 1 vs. 2 vs. 3) and Time as within-subject factor (T1 vs. T2), considering each SVIA subscale during mother–child feeding interactions as dependent variable.

Multivariate tests highlighted a significant effect played by Group (Wilks’s Lambda = 0.087, *F*_12,521.51_= 66.08, *p* = 0.000), Time (Wilks’s Lambda = 0.737, *F*_4,197_= 17.58, *p* = 0.000) and by the interaction between Group and Time (Wilks’s Lambda = 0.620, *F*_12,521.51_= 8.62, *p* = 0.000).

As far as it concerns the Group variable, univariate tests reported a statistically significant effect on all the SVIA subscales, i.e., on Maternal Affective State (*F*_3,200_= 438.63, *p* = 0.000), on Interactive Conflict (*F*_3,200_= 431.42, *p* = 0.000), on Food Refusal Behavior (*F*_3,200_= 391.31, *p* = 0.000) and on Dyad’s Affective State (*F*_3,200_= 457.79, *p* = 0.000). More specifically, Bonferroni *post hoc* testing revealed significant lower scores (*p* < 0.05) for Group 0 on all the SVIA subscales with respect to the other groups, whereas it reported significant higher scores (*p* < 0.05) four Group 1. As expected, Group 2 (the one with the mother diagnosed with BED) reported higher scores in all the SVIA subscales with respect to Group 3 (*p* < 0.05). Globally, Group 1 seemed to experience more difficulties during mother–child feeding interactions, followed, respectively, by Group 2, Group 3 and Group 0.

In regard to the variable Time, univariate tests reported a significant influence played on Maternal Affective State (*F*_1,200_ = 55.92, *p* = 0.000), on Interactive Conflict (*F*_1,200_= 41.09, *p* = 0.000), on Food Refusal Behavior (*F*_1,200_ = 63.84, *p* = 0.000) and on Dyad’s Affective State (*F*_1,200_ = 51.48, *p* = 0.000). More specifically, Bonferroni *post hoc* testing revealed a significant decrease (*p* < 0.05) in all the SVIA subscales during the transition from T1 to T2. Thus, the passing of time seemed to contribute in reducing difficulties during mother–child feeding interactions.

Finally, with respect to the interaction between Group and Time, univariate tests reported a significant effect on all the SVIA subscales, thus influencing the Maternal Affective State (*F*_3,2_ = 19.02, *p* = 0.000), the presence of Interactive Conflict (*F*_3,2_ = 13.93, *p* = 0.000) or of Food Refusal Behaviors (*F*_3,2_= 17.88, *p* = 0.000), and the Dyad’s Affective State (*F*_3,2_= 6.12, *p* = 0.001). More specifically, Bonferroni *post hoc* testing revealed specific changes for each group: Group 0 reported a significant decrease (*p* < 0.05) in all the SVIA subscales; Group 1 (both parents diagnosed with BED) reported a significant decrease (*p* < 0.05) in Food Refusal Behavior and Dyad’s Affective State; Group 2 (mother diagnosed with BED) showed a significant decrease (*p* < 0.05) in Interactive Conflict and in Dyadic Affective State; finally, Group 3 (father diagnosed with BED) showed a significant decrease (*p* < 0.05) in Maternal Affective State.

As regards mother–child feeding interactions, all the groups seemed to experience a significant decrease in the score of at least one SVIA subscale. Significant increases were never observed.

### Father–Child Feeding Interactions: Differences between Groups and Changes in Time

In order to investigate the presence of differences between the four groups in the quality of father–child feeding interactions and the presence of changes in time of such interactions, a mixed ANOVA was conducted on these data, with Group as between-subjects factor (0 vs. 1 vs. 2 vs. 3) and Time as within-subject factor (T1 vs. T2), considering each SVIA subscale during father–child feeding interactions as dependent variable.

Multivariate tests highlighted a significant effect played by Group (Wilks’s Lambda = 0.078, *F*_12,521.51_= 70.27, *p* = 0.000), by Time (Wilks’s Lambda = 0.781, *F*_4,197_= 13.79, *p* = 0.000) and by the interaction between Group and Time (Wilks’s Lambda = 0.514, *F*_12,521.51_= 12.41, *p* = 0.000).

In regard to the variable Group, univariate tests reported a statistically significant effect on all the SVIA subscales, i.e., on Father’s Affective State (*F*_3,200_= 455.03, *p* = 0.000), on Interactive Conflict (*F*_3,200_= 416.69, *p* = 0.000), on Food Refusal Behavior (*F*_3,200_= 416.56, *p* = 0.000) and on Dyad’s Affective State (*F*_3,200_= 537.21, *p* = 0.000). More specifically, Bonferroni *post hoc* testing revealed for Group 0 significant lower scores (*p* < 0.05) in all the SVIA subscales, with respect to the other groups, and reported for Group 1 significant higher scores (*p* < 0.05) on all the dimensions. With respect to Group 2 (mother diagnosed with BED), Group 3 (father diagnosed with BED) showed significant higher scores (*p* < 0.05) on all the SVIA subscales. In this sense, as expected, Group 0 was the one experiencing less difficulties during father–child feeding interactions, whereas such exchanges appeared more challenging in families where both parents or the father were diagnosed with BED.

In regard to the variable Time, univariate tests highlighted a statistically significant effect played on Father’s Affective State (*F*_1,200_ = 53.75, *p* = 0.000), on the presence of Interactive Conflict (*F*_1,200_= 31.46, *p* = 0.000) and of Food Refusal (*F*_1,200_ = 35.70, *p* = 0.000), and on Dyadic Affective State (*F*_1,200_ = 28.61, *p* = 0.000). More specifically, Bonferroni *post hoc* testing reported a significant decrease (*p* < 0.05) in all the SVIA subscales concerning father–child feeding interactions during the transition from T1 to T2.

Finally, with respect to the effect played by the interaction of Group and Time, univariate tests reported a statistically significant influence on Father’s Affective State (*F*_3,200_ = 28.60, *p* = 0.000), Interactive Conflict (*F*_3,200_ = 23.04, *p* = 0.000), Food Refusal Behavior (*F*_3,200_= 30.27, *p* = 0.000) and Dyad’s Affective State (*F*_3,200_= 21.24, *p* = 0.001). More specifically, Bonferroni *post hoc* testing revealed specific changes for each group: Group 0 (control) showed a significant decrease (*p* < 0.05) in all the SVIA subscales; Group 1 (both parents diagnosed with BED) showed a significant decrease (*p* < 0.05) in Father’s Affective State, Interactive Conflict and Dyadic Affective State; Group 2 exhibited a significant decrease (*p* < 0.05) in Father’s Affective State, Food Refusal and Dyadic Affective State; finally, for Group 3, a statistically significant increase (*p* < 0.05) was reported for Father’s Affective State, Food Refusal and Dyadic Affective State.

### Child’s Externalizing and Internalizing Symptoms: Differences between Groups and Changes in Time

In order to investigate the presence of differences between the four groups with respect to child’s symptoms and the presence of changes in time of such symptoms, mixed ANOVAs were conducted on these data, with Group as between-subjects factor (o vs. 1 vs. 2 vs. 3) and Time as within-subject factor (T1 vs. T2), considering the CBCL scores relative to the Externalizing and Internalizing symptomatology as dependent variables.

Multivariate tests reported a statistically significant effect played by Group (Wilks’s Lambda = 0.160, *F*_6,398_= 99.71, *p* = 0.000), Time (Wilks’s Lambda = 0.944, *F*_2,199_= 5.92, *p* = 0.000) and by the interaction between Group and Time (Wilks’s Lambda = 0.725, *F*_6,398_= 11.57, *p* = 0.000) on the summary scales.

In regard to the Group variable, univariate tests reported a statistically significant effect both on Internalizing (*F*_3,200_= 166.57, *p* = 0.000) and Externalizing Symptoms (*F*_3,200_= 248.08, *p* = 0.000).

Bonferroni *post hoc* testing highlighted for Group 0 (control) significantly lower scores both on the Internalizing and the Externalizing CBCL summary scales, when compared to the other groups. In regards to Internalizing Symptoms, no differences between Group 1 and Group 3 were found; in other words, children in the group where only the father was diagnosed with BED seemed to experience the same internalizing difficulties as shown by children whose both parents have been diagnosed with BED.

Regarding Externalizing Symptoms, Group 1 reported the highest scores (*p* < 0.05), followed by Group 3 (*p* < 0.05) and by Group 2 (*p* < 0.05). In this sense, children whose parents or whose father were diagnosed with BED seemed to exhibit a higher degree of externalizing difficulties.

In regards to the effect of the variable Time, univariate tests reported a significant influence only regarding Externalizing Symptoms (*F*_1,200_= 80.77, *p* = 0.030). More specifically, Bonferroni *post hoc* testing revealed a significant increase of externalizing symptomatology (*p* < 0.05) during the transition from T1 to T2.

Finally, with respect to the interaction between Group and Time, univariate tests reported a statistically significant effect both on Internalizing (*F*_3,200_ = 12.44, *p* = 0.000) and Externalizing Symptoms (*F*_3,200_= 12.93, *p* = 0.000).

More specifically, in regard to Internalizing Symptoms, Bonferroni *post hoc* testing revealed a significant decrease (*p* < 0.05) for Group 0 and Group 1, the absence of changes in Group 2 (*p* > 0.05) and a significant increase (*p* < 0.05) in the scores of Group 3. Regarding Externalizing Symptoms, Bonferroni *post hoc* testing reported a significant decrease (*p* < 0.05) in the scores of Group 0 and the absence of change in Group 1 (*p* > 0.05), whereas significant increases in externalizing scores were highlighted both for Group 2 and Group 3. In this sense, it appears that children with only one parent diagnosed with BED were more likely to display an increase in externalizing difficulties during the passing of time, while children of unselected populations or with both parents diagnosed with BED, instead, respectively, showed a decrease or stable level of externalizing symptoms.

### Model Assessment

The Pearson’s product-moment correlation coefficient was applied to maternal/paternal diagnosis (considered as present or absent), to the SVIA subscales during T1 and T2 and to the Internalizing and the Externalizing CBCL summary scales at T2 in order to test for associations between the presence of BED diagnosis in one of the parents, quality of feeding interactions and the intensity of internalizing and externalizing symptoms. **Table 6** reports the correlation matrix. Parental diagnosis of BED was correlated with all the measures considered. All the correlations were positive, indicating a direct association between the presence of diagnosis in one parent and the intensity of difficulties experienced during feeding interactions and regarding the children’s socio-emotional adjustment.

**Table 6:** Correlations between parental diagnosis, feeding interactions, and child’s internalizing/externalizing symptoms.

	1	2	3	4	5	6	7	8	9	10	11	12	13	14	15	16	17	18	19	20
1. Mother diagnosed with BED	-																			
2. Father diagnosed with BED	0.000	-																		
3. Maternal affective state (T1)	0.716^∗∗^	0.298^∗∗^	-																	
4. Interactive conflict (T1)	0.736^∗∗^	0.308^∗∗^	0.948^∗∗^	-																
5. Ch. Food refusal (T1)	0.744^∗∗^	0.263^∗∗^	0.936^∗∗^	0.928^∗∗^	-															
6. Dyadic affective state (T1)	0.739^∗∗^	0.373^∗∗^	0.956^∗∗^	0.952^∗∗^	0.938^∗∗^	-														
7. Father’s affective state (T1)	0.290^∗∗^	0.728^∗∗^	0.525^∗∗^	0.522^∗∗^	0.480^∗∗^	0.566^∗∗^	-													
8. Interactive conflict (T1)	0.313^∗∗^	0.733^∗∗^	0.537^∗∗^	0.545^∗∗^	0.489^∗∗^	0.572^∗∗^	0.937^∗∗^	-												
9. Ch. Food refusal (T1)	0.247^∗∗^	0.745^∗∗^	0.489^∗∗^	0.474^∗∗^	0.452^∗∗^	0.519^∗∗^	0.932^∗∗^	0.923^∗∗^	-											
10. Dyadic affective state (T1)	0.380^∗∗^	0.741^∗∗^	0.572^∗∗^	0.575^∗∗^	0.536^∗∗^	0.626^∗∗^	0.943^∗∗^	0.933^∗∗^	0.923^∗∗^	-										
11. Maternal affective state (T2)	0.840^∗∗^	0.432^∗∗^	0.744^∗∗^	0.770^∗∗^	0.764^∗∗^	0.794^∗∗^	0.564^∗∗^	0.576^∗∗^	0.536^∗∗^	0.642^∗∗^	-									
12. Interactive conflict (T2)	0.814^∗∗^	0.481^∗∗^	0.732^∗∗^	0.756^∗∗^	0.749^∗∗^	0.790^∗∗^	0.566^∗∗^	0.593^∗∗^	0.540^∗∗^	0.651^∗∗^	0.960^∗∗^	-								
13. Ch. Food refusal (T2)	0.837^∗∗^	0.411^∗∗^	0.736^∗∗^	0.753^∗∗^	0.749^∗∗^	0.779^∗∗^	0.553^∗∗^	0.575^∗∗^	0.529^∗∗^	0.631^∗∗^	0.943^∗∗^	0.944^∗∗^	-							
14. Dyadic affective state (T2)	0.822^∗∗^	0.463^∗∗^	0.758^∗∗^	0.774^∗∗^	0.771^∗∗^	0.812^∗∗^	0.596^∗∗^	0.601^∗∗^	0.557^∗∗^	0.671^∗∗^	0.948^∗∗^	0.940^∗∗^	0.948^∗∗^	-						
15. Father’s affective state (T2)	0.297^∗∗^	0.910^∗∗^	0.462^∗∗^	0.494^∗∗^	0.453^∗∗^	0.544^∗∗^	0.754^∗∗^	0.779^∗∗^	0.765^∗∗^	0.788^∗∗^	0.642^∗∗^	0.700^∗∗^	0.637^∗∗^	0.667^∗∗^	-					
16. Interactive conflict (T2)	0.377^∗∗^	0.854^∗∗^	0.480^∗∗^	0.517^∗∗^	0.461^∗∗^	0.561^∗∗^	0.736^∗∗^	0.761^∗∗^	0.741^∗∗^	0.782^∗∗^	0.681^∗∗^	0.733^∗∗^	0.684^∗∗^	0.715^∗∗^	0.956^∗∗^	-				
17. Ch. Food refusal (T2)	0.302^∗∗^	0.894^∗∗^	0.462^∗∗^	0.493^∗∗^	0.451^∗∗^	0.539^∗∗^	0.748^∗∗^	0.768^∗∗^	0.758^∗∗^	0.788^∗∗^	0.646^∗∗^	0.698^∗∗^	0.634^∗∗^	0.679^∗∗^	0.955^∗∗^	0.955^∗∗^	-			
18. Dyadic affective state (T2)	0.304^∗∗^	0.897^∗∗^	0.457^∗∗^	0.486^∗∗^	0.437^∗∗^	0.536^∗∗^	0.739^∗∗^	0.761^∗∗^	0.749^∗∗^	0.776^∗∗^	0.638^∗∗^	0.700^∗∗^	0.640^∗∗^	0.661^∗∗^	0.963^∗∗^	0.947^∗∗^	0.956^∗∗^	-		
19. Internalizing symptoms (T2)	0.230^∗∗^	0.598^∗∗^	0.266^∗∗^	0.308^∗∗^	0.232^∗∗^	0.312^∗∗^	0.431^∗∗^	0.480^∗∗^	0.466^∗∗^	0.490^∗∗^	0.474^∗∗^	0.535^∗∗^	0.484^∗∗^	0.468^∗∗^	0.689^∗∗^	0.716^∗∗^	0.710^∗∗^	0.728^∗∗^	-	
20. Externalizing symptoms (T2)	0.375^∗∗^	0.746^∗∗^	0.457^∗∗^	0.464^∗∗^	0.417^∗∗^	0.495^∗∗^	0.613^∗∗^	0.641^∗∗^	0.627^∗∗^	0.648^∗∗^	0.646^∗∗^	0.688^∗∗^	0.652^∗∗^	0.653^∗∗^	0.822^∗∗^	0.850^∗∗^	0.828^∗∗^	0.843^∗∗^	0.776^∗∗^	-

*^∗∗^Correlation is significant at the 0.01 level (2-tailed).*

Given the presence of such associations a path analysis model was created in order to investigate the role played by feeding interactions (both with the mother and with the father) as mediators on the effect of parental diagnosis on the child’s socio-emotional adjustment. The model was tested using LISREL 8.80 (Jöreskog and Sörbom, 2006), which introduces the possibility to consider complex sets of relationships in a simultaneous fashion. The procedure provides path coefficients as part of the model results, i.e., parameter estimates of the relative effect of one variable on another. Standardized regression weights β indicate the strength of the linear relation and imply a direct relation between changes in the connected variables. Moreover, to assess the overall fit of the data to the model, the LISREL procedure also provides chi-square values, goodness-of-fit indices and squared multiple correlations. The chi-square assessment of fit refers to the possibility for a hypothesized model to adequately fit the data. Goodness-of-fit indices range from 0 to 1 with values close to 1 indicating good fit. Squared multiple correlations are indications of the amount of variability accounted for by the given equation.

**FIGURE 1:**
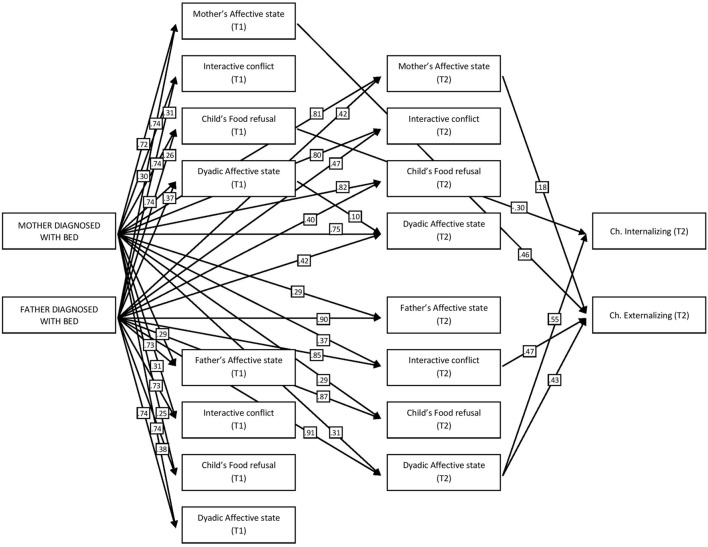
**Path model significant parameter estimates for the effects of parental diagnosis on feeding interactions and on the child’s internalizing and externalizing symptoms (significant *p* < 0.05)**.

The chi-square value for the entire model was 270.45 (df = 96, *p* = 0.01) which was an acceptable result (Schermelleh-Engel et al., 2003). Regarding the goodness-of-fit indices, the Non-normed Fit index (NNFI) and the Comparative Fit Index (CFI) were 0.97 and 0.99, respectively. The high level of both indices indicated good fit of the model to the actual data. The value for the Root Mean Square Error of Approximation (RMSEA) instead was higher than expected (RMSEA = 0.095). Usually, in fact, criteria in the range [0.01–0.8] have been proposed to indicate an excellent to acceptable fits (Schermelleh-Engel et al., 2003). Other authors have suggested the value 0.1 as the higher cut-off to employ a certain model (Browne and Cudeck, 1992). Moreover, recent literature criticized the use of fixed cut-off points in RMSEA test statistics on the basis of their lack of empirical support (Chen et al., 2008). Given these reasons, and also considering the acceptability of the other indices, the model was judged globally adequate to fit the data. **Figure 1** gives the statistically significant standardized structural parameter estimates for the model, suggesting the direct and indirect effects of maternal and paternal diagnosis of BED on the child’s internalizing and externalizing symptoms. As it is possible to see, having a parent (either the mother or the father) diagnosed with BED significantly affected feeding interactions, with both parents during the two periods considered (*p* < 0.05). This influence was direct, and it involved all the SVIA variables both during T1 and T2. Moreover, the model confirmed the presence of an indirect effect played by the parental diagnosis of BED on child socio-emotional adjustment. During mother–child feeding interactions this indirect effect seemed to act through the Mother’s affective state, both during T1 and T2; more specifically, the Mother’s affective state during T1 (β = 0.46, *p* < 0.05) and during T2 (β = 0.18, *p* < 0.05) played a statistically significant effect on Externalizing symptoms, whereas the child’s Food refusal behaviors during T1 mediated the effect played by parental diagnosis on Internalizing symptoms (β = -0.30, *p* < 0.05). On the other hand, during father–child interactions, the effect of parental diagnosis on Externalizing symptoms was mediated by the Interactive conflict during T2 (β = 0.47, *p* < 0.05) and the Dyad’s affective state during T2 (β = 0.43, *p* < 0.05). Finally, the Dyad’s affective state during T2 also acted as a mediator on the effect of BED diagnosis on Internalizing symptoms (β = 0.55, *p* < 0.05).

**FIGURE 2:**
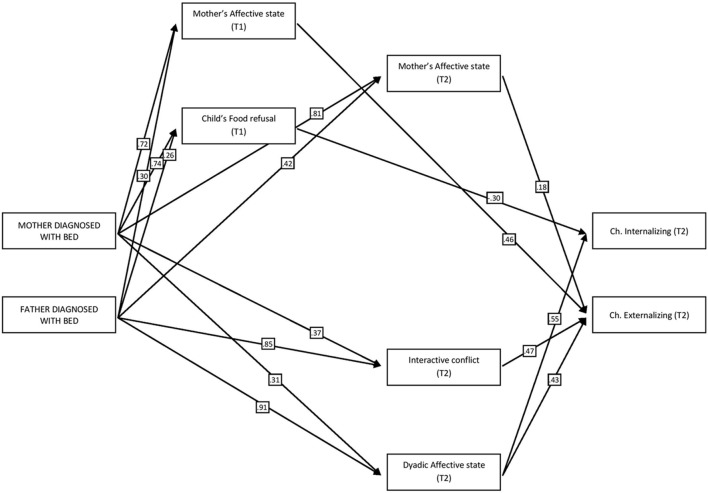
**Significant indirect paths linking parental diagnosis to the child’s internalizing/externalizing symptoms (Significant *p* < 0.05)**.

Given the numerous variables, in order to achieve a clearer view of the mediated effects of parental diagnosis, a **Figure 2** was inserted containing only the significant indirect paths linking maternal/paternal diagnosis to the child’s Internalizing/Externalizing symptoms. As it is possible to see, the most important mediator during mother–child interactions was Maternal affective state, which was significant both during T1 and T2, whereas for father–child interactions the indirect effect of parental diagnosis seemed to begin later in time (T2) and was mainly conveyed through more interactive variables, such as Interactive conflicts and the Dyad’s affective state. Furthermore, the indirect effect of paternal diagnosis was higher both for Internalizing (β = 0.64, *p* < 0.05) and Externalizing symptoms (β = 0.73, *p* < 0.05) with respect to the effect of maternal diagnosis (β = 0.24, *p* < 0.05 for internalizing symptoms and β = 0.38, *p* < 0.05 for externalizing symptoms).

## Discussion

The main aim of this paper was to assess whether the quality of interactive exchanges during feeding between parents with BED and their children might affect the expression of offspring’s internalizing and externalizing symptoms. Through an observation procedure (SVIA), we longitudinally studied mother–infant and father–infant interactions during feeding at 18 (T1) and 36 months of the child (T2) verifying possible significant differences in four groups: Group 0 consisted of healthy controls; Group 1 included both parents diagnosed with BED, Group 2 had mothers diagnosed with BED, Group 3 contained fathers diagnosed with BED.

Overall, Group 1 showed significantly higher scores than all others at SVIA. Our data indicate that, in this group, both mothers and fathers presented more maladaptive relational exchanges with their offspring during feeding, as compared to other groups. This result indicates that the moment of feeding for parents with BED and their children is particularly challenging, and the dyads (both mother–child and father–child) are characterized by unattuned interactions, lack of parental sensitivity, and a general negative emotional climate. Previous literature in the field had demonstrated that mothers with eating disorders and their children show problematic interactions during feeding (Stein et al., 2013). Yet, this study adds to previous studies because it addresses the specific effects of BED in parents and the characteristics of father–infant exchanges. Moreover, it does so using an observational measure, whereas self-report or report-form questionnaires filled-out by parents have largely been used in previous studies (Cerniglia et al., 2014a). It is noteworthy that group 1 scores at SVIA remained significantly higher than those of other groups at the second assessment point (T2), indicating that, consistent with Fassino et al.’s (2009) studies in a sample without treatment, their risk of relational difficulties do not spontaneously reduce over time.

The groups where only one parent were diagnosed for BED (Group 2 and Group 3) showed more maladaptive scores at SVIA than the control group. This result suggests that, while the presence of both parents with BED is highly associated with the development of problematic mother–child and father–child exchanges during feeding, the families where only one parent was diagnosed were still at significantly higher risk of being characterized by difficulties in undertaking fluent feeding routines with their offspring.

Though, as stated above, maladaptive interactions remain higher over time whereas group 0, 2, and 3, maternal SVIA scores decrease in all groups from T1 (18 months of the child) to T2 (36 months of the child). That is to say that while families with both parents diagnosed with BED maintain the highest risk of having problematic interactions with their children, these difficulties decrease in mother–infant dyads if the whole sample is considered. The dyads with fathers with BED (group 3) do not show reduced maladaptive relational patterns at T2.

Further studies, which must also consider attachment experiences as possible predictors of adaptive or maladaptive development in children, are needed to clarify this point, but we make the hypothesis that there may be a reciprocal adjustment operated by the child to the mother’s psychopathology and relational difficulties (which impact on the quality of interactional patterns during feeding; Cimino et al., 2016) and by the mothers to possible individual problematic characteristics of the child, such as, for example, difficult temperament or specific sensory aversion to some foods (Romano et al., 2015). These two adaptation processes are probably reinforced by the improved individual capacities of the child of eating without being fed by the mother (at T2; 36 months of age of the child), which may reduce her emotional overload. These processes seem not to occur in families where only the fathers were diagnosed with BED (Group 3). It is possible that as emotional pressures reduce for mothers, this is experienced as increasingly burdensome for fathers, during this period of child development that appears to be associated with a general increase in paternal involvement in offspring feeding (Lamb and Lewis, 2013).

After assessing parent–infant interactions during feeding, we studied the presence and the stability or change of internalizing and externalizing symptoms of the children at T1 and T2 in the four groups.

We found that children in group 1 (both parents with BED) showed significantly higher internalizing and externalizing symptoms, when compared to offspring in other groups and group 0 showed significantly lower scores than all others both at T1 and T2. Over time, externalizing problems significantly increased in children belonging to groups where only the mother or only the father had BED. Offspring of fathers with BED showed increased internalizing symptoms from T1 to T2. Although other studies have widely demonstrated that internalizing or externalizing problems in children of psychiatrically diagnosed parents tend to increase over time, in the absence of any treatment (Shanahan et al., 2014), this is the first research to report detailed results, specifically for children of fathers and mothers with BED. While the presence of both parents with BED diagnosis was a factor associated with more maladaptive feeding interactions, this “double risk” seems not to affect the severity of children’s internalizing or externalizing symptoms.

Our further aim was to assess the possible role of the quality of parent–infant interactions during feeding in predicting offspring’s internalizing and externalizing symptoms in Groups 1–3.

Consistent with the Development Psychopathology theoretical framework (Davies and Cicchetti, 2004), according to which the transmission of psychopathological risk is regulated from parents to children both by individual and interactional factors, we created a predictive model aimed at assessing the specific role of the quality of feeding interactions as mediators of the effects of parental BED diagnosis on their offspring’s internalizing and externalizing symptoms. Our model confirmed a direct influence of parental psychiatric diagnosis on the quality of mother–infant and father–infant interactions, both at T1 and T2. Moreover, our results confirmed that dyadic feeding interactions mediate the influence of parental diagnosis on children’s psychological functioning.

It is noteworthy that our data show different subscales of SVIA (that is different dimensions composing the general quality of parent–child feeding interactions) to mediate the effect of parental diagnoses. Maternal Affective State mediates the effect of mothers’ diagnosis on children’s externalizing problems (both at T1 and T2), whereas child’s Food Refusal Behavior mediates the influence of mothers’ BED on their offspring’s internalizing symptoms. In the case of fathers, however, the mediating effect of the quality of feeding interactions is specifically expressed in the characteristics of Dyadic Affective State and Interactive Conflict at T2. These results indicate that while the direct weight of parental diagnosis is strong in predicting maladaptive outcomes in children, the quality of interactions during routine activities, which include affective and behavioral exchanges is crucial in shaping specific psychological profiles in children (i.e., the expression of internalizing or externalizing symptoms) and their development over time. Moreover, our results suggest that the quality of interactions with their fathers during feeding assumes a mediating role only at 36 months of age of the children. This is consistent with Trautmann-Villalba et al.’s (2006) studies, which demonstrated how the quality of mother–child interactions in the first months of life is an essential predictor of offspring’s adaptive or problematic psychological functioning, whereas the characteristics of father–child exchanges appear to influence offspring functioning (Cerniglia et al., 2014b).

This study has some limitations. First, we used report-form questionnaire to assess internalizing and externalizing children’s symptoms. Observational and/or more objective measures are needed to minimize the risk of distortions in parents’ perception of their offspring’s psychological functioning. Second, we did not evaluate the severity of parental psychopathology, which could influence the severity and form of children’s symptoms. Third, the homogeneity of the sample, in terms of cultural, geographical, and socio-economic status, limits replication of the study in other countries or cultures. Finally, statistical controls were not applied for potential confounders such as child abuse or neglect.

Notwithstanding the above limitations, the present study adds to the previous literature in several ways.

This is the first study, to our knowledge, to recruit families where both parents showed the same psychiatric diagnosis (and specifically the BED diagnosis, which has only recently been included in DSM-5), giving detailed results on how maternal and paternal diagnoses (or the conjunct risk of the presence of both parents’ diagnoses) differently influence their offspring’s internalizing and externalizing symptoms.

Further, the quality of parent–child interactions was assessed through an observational method, administered by mental health clinicians specifically trained in the use of the measure and for the aims of this study. Lastly, this was a longitudinal study, which investigated both continuity and change in variables across two assessment points (18 and 36 months of age) for families with parents with BED, thereby representing an important development in the field.

## Author Contributions

SC prepared the study design and supervised the research team; LC wrote the introduction section of the manuscript and recruited the sample; AP prepared data set, performed statistical analyses and prepared tables and figures; AS wrote the discussions section of the manuscript; LR performed path analyses and supervised statistical analyses; GB recruited the sample and wrote the references. All authors reviewed the manuscript.

## Conflict of Interest Statement

The authors declare that the research was conducted in the absence of any commercial or financial relationships that could be construed as a potential conflict of interest.
